# Inhibition of coiled coil domain containing protein 69 enhances platinum-induced apoptosis in ovarian cancer cells

**DOI:** 10.18632/oncotarget.21356

**Published:** 2017-09-28

**Authors:** Long Cui, Bo Liang, Yihua Yang, Minhui Zhu, Joseph Kwong, Hongliang Zheng, Chi Chiu Wang

**Affiliations:** ^1^ Department of Otorhinolaryngology-Head and Neck Surgery, Changhai Hospital, Second Military Medical University, Shanghai, People's Republic of China; ^2^ Department of Obstetrics and Gynaecology, Prince of Wales Hospital, The Chinese University of Hong Kong, Shatin, Hong Kong; ^3^ Center of Reproductive Medicine, Affiliated Hospital of Guilin Medical College, Guilin, People's Republic of China; ^4^ Reproduction and Development Laboratory, Li Ka Shing Institute of Health Sciences, The Chinese University of Hong Kong, Shatin, Hong Kong; ^5^ School of Biomedical Sciences, The Chinese University of Hong Kong, Shatin, Hong Kong

**Keywords:** cell cycle arrest, chemoresistance, ovarian cancer, apoptosis, p53 acetylation

## Abstract

Cisplatin is a platinum-based drug that is used for the treatment of human gynecological cancers. However, molecular mechanisms of chemo-resistance in ovarian cancer are poorly understood. The aim of the study is to examine the role of coiled coil domain containing protein 69 (CCDC69) in the underlying mechanism of chemoresistance. Heavy CpG methylation (73.1% and 74.3%) was found in A2780 and A2780cis cells assessing by bisulfite sequencing. Restoration in the expression of CCDC69 was found in A2780 and A2780cis cells after 5-Aza-dC treatment. In fact, the expression levels of CCDC69 were about 3-4 fold higher in cisplatin-resistant A2780cis cells than its parental cisplatin-sensitive A2780 cells. When knockout CCDC69 in cisplatin-resistant A2780cis and SKOV3 cells by CRISPR/Cas9, the CCDC69 knockout cisplatin-resistant A2780cis and CCDC69 knockout SKOV3 cells were also shown increased sensitive to cisplatin treatment. Moreover, treating CCDC69 knockout A2780cis cells with cisplatin, abrogated G1 and G2/M arrest, increased of cleaved caspase 3&8, greater ΔΨm loss and higher levels of Bax were observed. When restoring CCDC69 expression in CCDC69 knockout A2780cis cells by transient transfection, it attenuated sensitivity to cisplatin. By immunoblotting, we found that depletion of CCDC69 increased p53 acetylation at K382 site and Bax mitochondrial redistribution. Additionally, inhibition of c-Myc enhanced cisplatin sensitivities in CCDC69 knockout A2780cis cells, overexpression of c-Myc reduced apoptosis in CCDC69 knockout SKOV3 cells. Our results showed that CCDC69 inhibition might interfere with the effectiveness of combination therapy with platinum drugs.

## INTRODUCTION

Epithelial ovarian cancer (EOC) is one of the leading cause of death among women worldwide [1]. 70% of the patients relapse within 5 years following the current standard chemotherapy of systemic platinum and taxane-based medication before or after aggressive surgical cytoreduction [2]. Many patients develop chemoresistance after several courses of the treatment, contributing to poor outcome in late-stage of epithelial ovarian cancer [3]. Hence, there is an unmet medical need to know the mechanism conferring chemoresistance and identify novel therapeutic targets to improve the clinical outcomes.

Cisplatin, a component of standard platinum-based treatment regimens for EOC, forms adduct with cellular nucleophiles within cancerous cell DNA. The molecular mechanism of chemoresistance to platinum compounds are related to multiple factors. It involves increased efflux of platinum compounds resulting from levels of glutathione, decreased platinum agents uptake and enhanced DNA repair [4, 5]. Cisplatin activates the DNA damage response pathway, in which tumor suppressor gene p53 controls cell cycle progression and apoptosis [6, 7]. It was evidenced that p53 induces either G1 or G2 arrest by inducing a variety of expression of its downstream target genes such as p21, GADD45 and 14-3-3 [8].

Although the definite DNA damage mechanism caused by platinum agents remains unknown, the irreversible DNA damage often leads to activation of apoptotic pathway [9, 10]. The cisplatin resistance may result from its inability to induce apoptosis [11, 12]. DNA damage initiates apoptosis through p53 transcription-dependent and independent mechanisms [13, 14]. P53 transcriptionally activates expression of Bcl-2 pro-apoptotic proteins such as Bax, Noxa and Puma, eventually leading to mitochondrial cell death [15]. Alternatively, p53 induces transcription-independent apoptosis through its translocation to the mitochondria and triggers mitochondrial outer membrane potential (ΔΨm) by Bax oligomerization [16]. Post-translational modifications such as acetylation influence the stability of p53 [17, 18]. For instance, in response to genotoxic stress, acetylation at K320/K373/K382 of p53 activates pro-apoptotic functions of Bax [19]. Acetylation of p53 at K382 and K381 in various cancer cell lines leads to induction of Puma and cell death [20].

Coiled-coil domain-containing (CCDC) proteins are involved in diverse regulatory functions related to their highly versatile coiled-coil motif [20, 21]. CCDC69 has been identified as a regulator for DNA replication and formation of mitotic spindle in eukaryotic cells [22]. Downregulation of CCDC69 forms aberrant central spindles and leads to dislocation of Aurora B [21]. Aurora kinases B is involved in maintenance of centrosome function, bipolar spindle assembly, and chromosome segregation [22]. Overexpression of Aurora kinase B has been detected in a variety of malignant tumors and is found to be correlated with poor overall survival [23]. Moreover, the efficacy of cisplatin in resistant ovarian cancer cells was enhanced when combined with Aurora kinases B inhibitors [24–26]. However, little functional study was performed to study the function of CCDC69 in the chemoresistance of ovarian cancer.

In the present study, we initially observed that CCDC69 expression was significantly elevated in the cisplatin-resistant A2780cis cells than the cisplatin-sensitive A2780 cells. We then identified stable CCDC69 knockout A2780cis and SKOV3 cells were more sensitive to cisplatin than their corresponding wildtype cells. Furthermore, we found inhibition of CCDC69 expression abrogated the G1 and G2/M arrest. Our results also showed that exposure to cisplatin after depletion of CCDC69 increased mitochondrial injury and upregulated p53 acetylation. In addition, the cisplatin induced cytotoxicity is related to the modulation of c-Myc protein expression.

## RESULTS

### *CCDC69* expression is upregulated in cisplatin-resistant ovarian cancer cells

**Figure 1:**
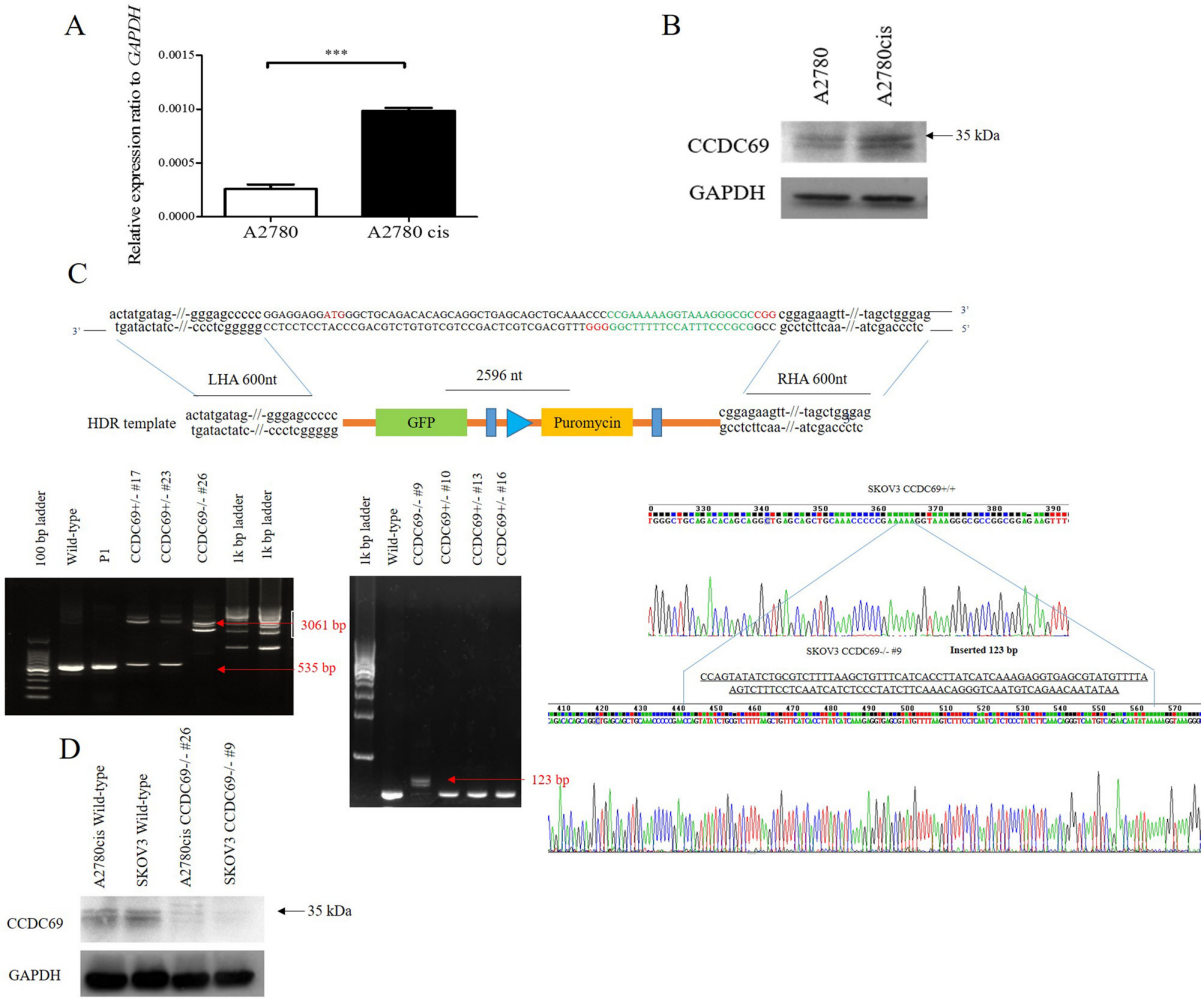
Overexpression of *CCDC69* in A2780cis cells and generation of CCDC69 knockout A2780cis and SKOV3 cells using the CRISPR/Cas9 system **(A)** The relative expression level of *CCDC69* was elevated in A2780cis cells compared with A2780 cells by Real-time RT-PCR. GAPDH was used as a loading control. Data represent the mean and the standard deviation from three independent experiments. ^***^p < 0.001 versus A2780 cells (Student's t-test). **(B)** The level of CCDC69 protein was overexpressed in A2780cis cells compared with A2780 cells by western blot analysis. The upper band marked as around 35 kDa as arrow indicated. Detection of GAPDH was used as a loading control. **(C)** Upper panel: Schematic representation of the *CCDC69* genomic region, targeted PAM positions, and primer positions. Knockout *CCDC69* allele containing the GFP-puromycin cassette. PAM, protospacer adjacent motif. HDR, homologous directed repair. LHA, left homologous arm. RHA, right homologous arm. Black arrows indicate primer positions. Lower panel: Two CCDC69-knockout cell lines were established from A2780cis and SKOV3 cells, respectively. The CCDC69 genomic region was analyzed by PCR. Amplicons were separated in agarose gels. Using the 69F + 69R primer set, the 535 bp wild type region was amplified in A2780cis and SKOV3 cells, whereas longer amplicons were detected in CCDC69-knockout cell lines. A2780cis CCDC69-/- #26 cells have insertions of 2596 bp and SKOV3 CCDC69-/- #9 cells have insertions of 123 bp. Red arrows indicate successful donor oligonucleotide genomic integration. **(D)** CCDC69 protein levels in the indicated cell lines. CCDC69 protein expression was analyzed by Western blot analysis. GAPDH was used as the loading control.

To know if *CCDC69 plays* a crucial role of in cisplatin resistance, we assessed the difference in expression of *CCDC69* between cisplatin-resistant ovarian cancer cells A2780cis and cisplatin-sensitive ovarian cancer cells A2780 by real-time quantitative PCR and western blotting. Upregulation of CCDC69 mRNA expression (3.9-fold increase, p < 0.0001; Figure 1A) was found in cisplatin-resistant A2780cis cells compared with cisplatin-sensitive A2780 cells, and the CCDC69 protein expression increased 2.4-fold in cisplatin-resistant A2780cis cells (p < 0.01; Figure 1B).

### Knockout CCDC69 in chemo-resistant ovarian cancer A2780cis and SKOV3 cells by CRISPR/Cas9

To evaluate the functions of *CCDC69* in the cisplatin resistance, we generated stable CCDC69 knockout A2780cis and SKOV3 cells by CRISPR/Cas9 technology. We designed two gRNAs against exon 1 of *CCDC69* and primers amplifying the targeted *CCDC69* genomic region. Sequence analysis of the PCR products revealed that A2780cis CCDC69-/- #26 cells had 2596 bp insertions and SKOV3 CCDC69-/- #9 cells had 123 bp insertions (Figure 1C), representing two independent CCDC69 knockout A2780cis cells (referred as A2780cis CCDC69-/- #26) and CCDC69 knockout SKOV3 cells (referred as SKOV3 CCDC69-/- #9), respectively. Western blotting results confirmed that almost no CCDC69 protein expression was observed in A2780cis CCDC69-/- #26 and SKOV3 CCDC69-/- #9 cells (Figure 1D).

### Depletion of CCDC69 in ovarian cancer cells enhanced cisplatin induced-apoptosis

**Figure 2:**
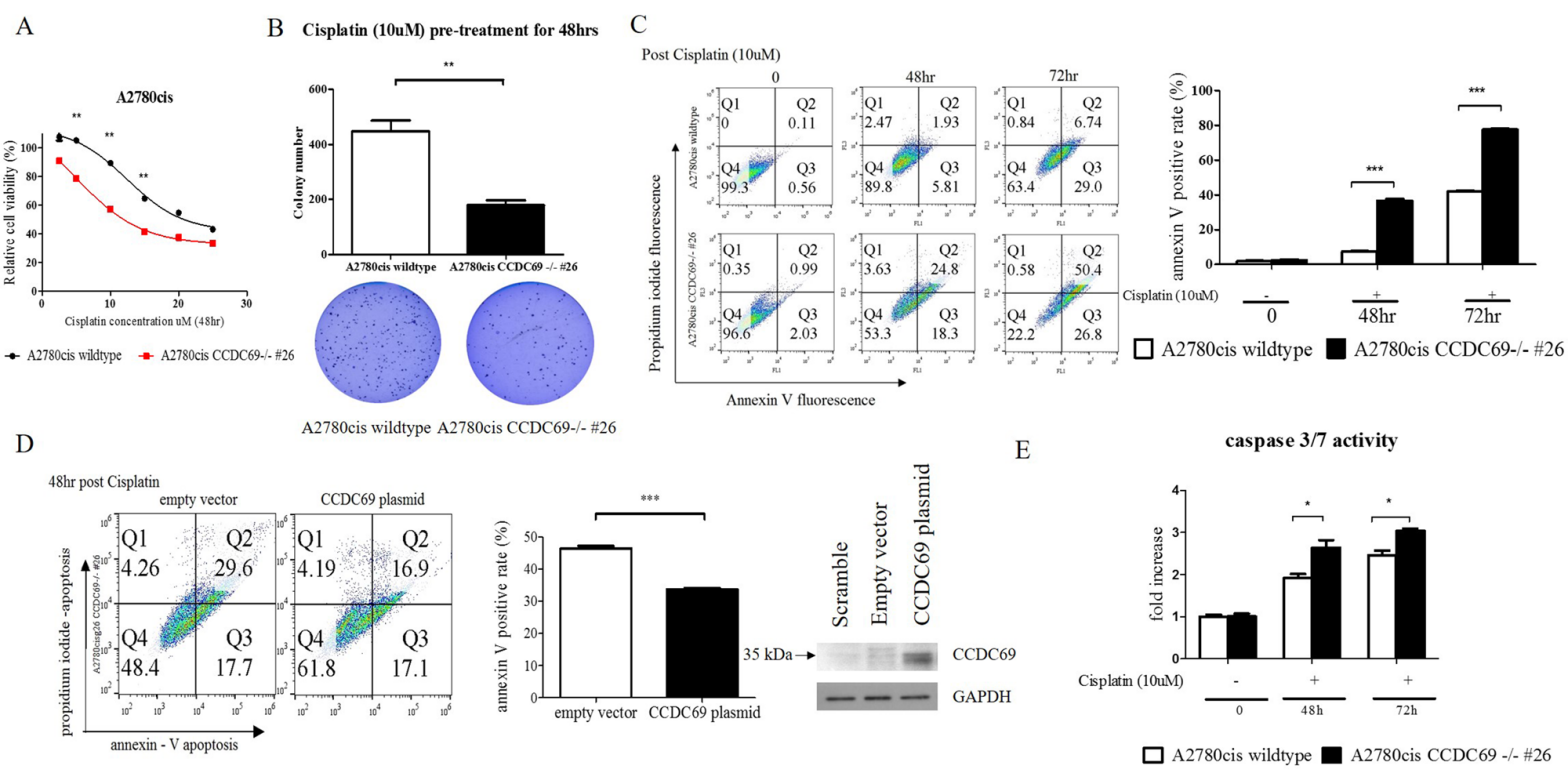
Depletion of *CCDC69* in A2780cis ovarian cancer cells enhanced cisplatin induced-apoptosis **(A)** Sensitization of cells to cisplatin after CCDC69 knockout as revealed by the CCK-8 cytotoxicity assay. **(B)** Number of colonies were significantly decreased in A2780cis CCDC69-/- #26 cells as revealed by colony formation assays. The colonies were counted using Image J software. **(C)** Apoptosis was analyzed by flow cytometry after annexin V and propidium iodide staining. Cells were incubated with and without cisplatin (10 uM) for 48 hr and 72 hr. Total apoptosis is the sum of the percentage of annexin V only and annexin V/propidium iodide stained cells. **(D)** Confirmation of CCDC69 expression in A2780cis CCDC69-/- #26 cells transfected with plasmids expressing the indicated CCDC69 protein. Stained annexin V of the transfected cells after treatment with cisplatin were assessed by flow cytometry. **(E)** Increase cleavage of caspase 3/7 activity after treatment with cisplatin.

**Figure 3:**
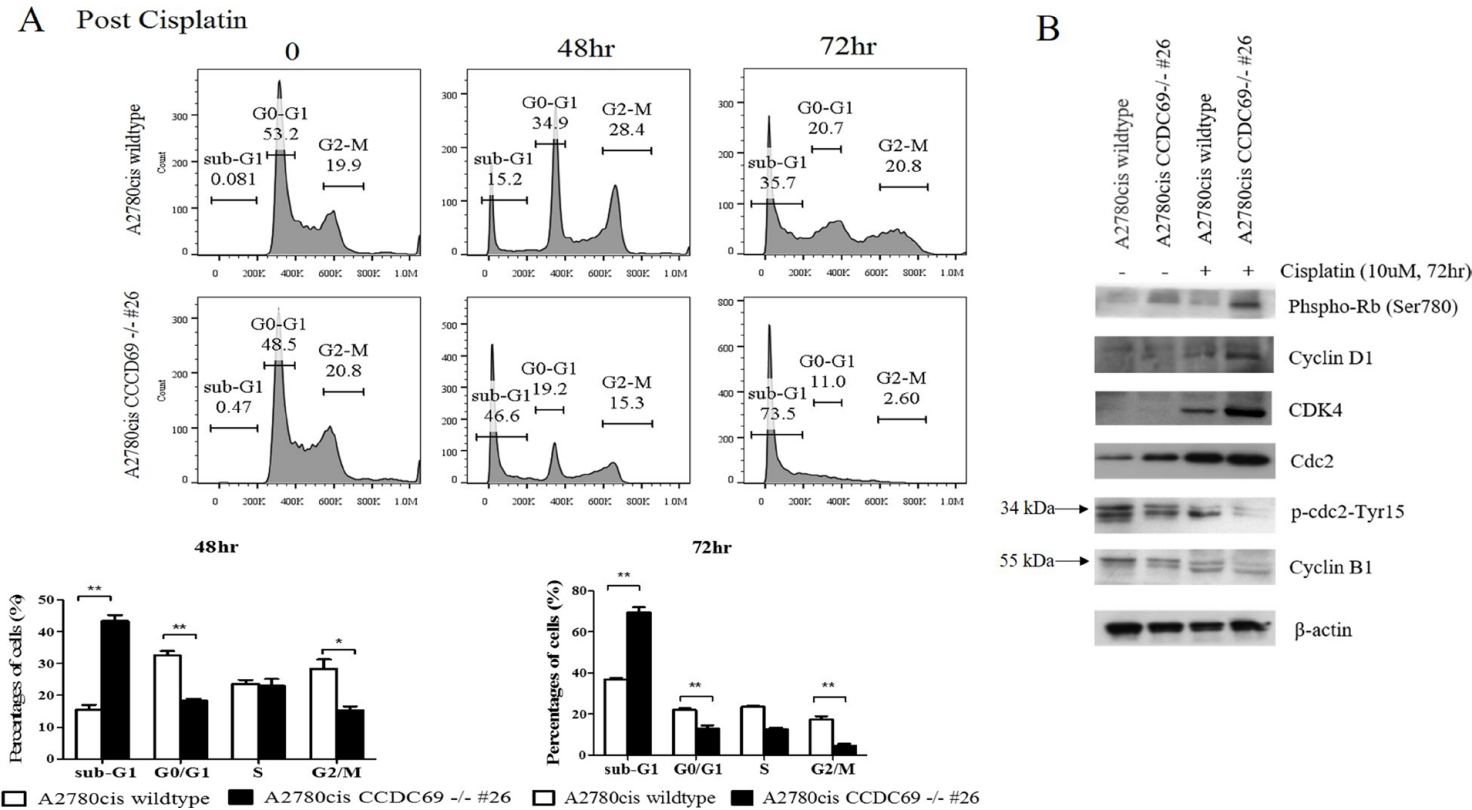
*CCDC69* depletion abolished G1 and G2/M arrest after cisplatin treatment **(A)** A2780cis wildtype and A2780cis CCDC69 -/- #26 cells were treated with 10 μM cisplatin for 48 hr and 72 hr, then cell cycle was analyzed by flow cytometry. Data represent the mean and the standard deviation from three independent experiments. ^*^p < 0.05 and ^**^p < 0.01 versus cisplatin-treated A2780cis wildtype cells (Student's t-test). **(B)** Immunoblot detection of effect of depletion of *CCDC69* on the G1 and G2/M phase related protein levels in A2780cis wildtype and A2780cis CCDC69 -/- #26 cells before and after cisplatin treatment.

To understand the effect of CCDC69 knockout on cisplatin sensitivity, cell viability was examined by CCK-8 cytotoxicity assay after treatment with a series of concentrations of cisplatin. A2780cis CCDC69-/- #26 cells had 2.5-fold lower IC50 of cisplatin than A2780cis wildtype cells (p <0.001) (Figure 2A), suggesting that depletion of *CCDC69* resensitized the cisplatin-induced cytotoxicity in the cisplatin-resistant ovarian cancer cells. Pre-treatment with cisplatin at 10 μM for 48 hr significantly decreased the amount of colonies after 14 days in A2780cis CCDC69-/- #26 cells than A2780cis wildtype cells (p<0.001, Figure 2B). Annexin V positive cells were significantly increased in the A2780cis CCDC69-/- #26 cells treating with cisplatin compared with A2780cis wildtype cells (p<0.0001, Figure 2C). Consistent with annexin V/PI staining data, A2780cis CCDC69-/- #26 cells showed a significant higher loss of mitochondrial transmembrane potential than A2780cis wildtype cells after treatment with cisplatin using JC-1 staining (Supplementary Figure 1A). PI staining and sub-G1 cell cycle analysis further confirmed that depletion of *CCDC69* in A2780cis cells significantly increased the percentage of apoptotic cells followed by cisplatin treatment (Figure 3A). To further confirm that CCDC69 increased sensitivity to cisplatin, we transiently transfected either an empty vector plasmid or a CCDC69-expressing construct into the stable CCDC69 knockout cells, followed by treatment with cisplatin. As expected, transfection of the CCDC69 expression construct into CCDC69 knockout cells significantly decreased apoptosis compared to transfected with empty vector as measured by annexin V staining, in presence of cisplatin (Figure 2D). Increased expression levels of active caspase-3/7 and cleaved PARP was detected in CCDC69 Knockout A2780cis cells (Figure 2E and Supplementary Figure 1B). To identify the apoptotic pathway that is activated after knockout of CCDC69, cleavage of caspase-8 was also assessed (Supplementary Figure 1C). The cleavage of both caspase-8 and loss of mitochondrial transmembrane potential indicated that both intrinsic and extrinsic apoptosis pathways were activated. Knockout of CCDC69 also induced cisplatin-induced cytotoxicity and apoptosis in SKOV3 ovarian cancer cells (Supplementary Figure 2). Collectively, these findings clearly indicate that CCDC69 is crucial for cisplatin-induced apoptosis in ovarian cancer cells.

### Depletion of CCDC69 abrogated G1and G2/M cell cycle arrest in ovarian cancer cells following treatment with cisplatin

FACS analysis data showed that cisplatin-treated A2780cis CCDC69^-/-^ #26 cells had lower percentage of G0/G1(p < 0.01) and G2/M phase (p < 0.05) followed by obvious aggregation of these cells at sub-G1 than that of cisplatin-treated A2780cis wildtype cells (Figure 3A). These results suggested that CCDC69 depletion abrogated cisplatin-induced cell cycle arrest in induction of apoptosis by cisplatin. Interestingly, carboplatin treated A2780cis CCDC69^-/-^ #26 cells only has lower percentage of G2/M phase when compared carboplatin treated A2780cis wildtype cells (p < 0.05, Supplementary Figure 3).

The flow cytometric data demonstrated that cisplatin-resistant A2780cis wildtype cells accumulated in G1 and G2 phase treating with cisplatin and before undergoing apoptosis, whereas A2780cis CCDC69^-/-^ #26 cells abrogated in these two phases (Figure 3A). Cell cycle regulators mediate cisplatin resistance has been shown in other studies [29]. Here, we investigated whether expression of key cell cycle regulatory proteins might be altered in A2780cis wildtype and A2780cis CCDC69-/- #26 cells in response to cisplatin. As shown in Figure 3B, in response to cisplatin treatment, the protein expression levels of CDK4, cyclin D1 and Phospho-Rb (Ser780) in A2780cis CCDC69-/- #26 cells were remarkably increased compared with A2780cis wildtype cells (Figure 3B). Additionally, we next assessed the protein expression levels responsible for G2 progression such as Cdc2 and cyclin B1 following treatment with cisplatin. A striking up-regulation of Cdc2 expression was detected in cisplatin treated A2780cis CCDC69-/- #26 cells (Figure 3B). Since phosphorylation Tyr15, resulting in inhibition of Cdc2, we tested the Tyr15 phosphorylation status of Cdc2. Figure 3B showed that Tyr15 phosphorylated Cdc2 was apparently decreased in the A2780cis CCDC69-/- #26 cells. Furthermore, cyclin B1 protein level was significantly downregulated in A2780cis CCDC69-/- #26 cells than wildtype A2780cis cells. Collectively, these results indicate that depletion of CCDC69 abrogated cell cycle arrest through regulating cell cycle progression proteins.

### Expression of p53, acetyl-p53-382 and Bax after *CCDC69* depletion following cisplatin treatment

**Figure 4:**
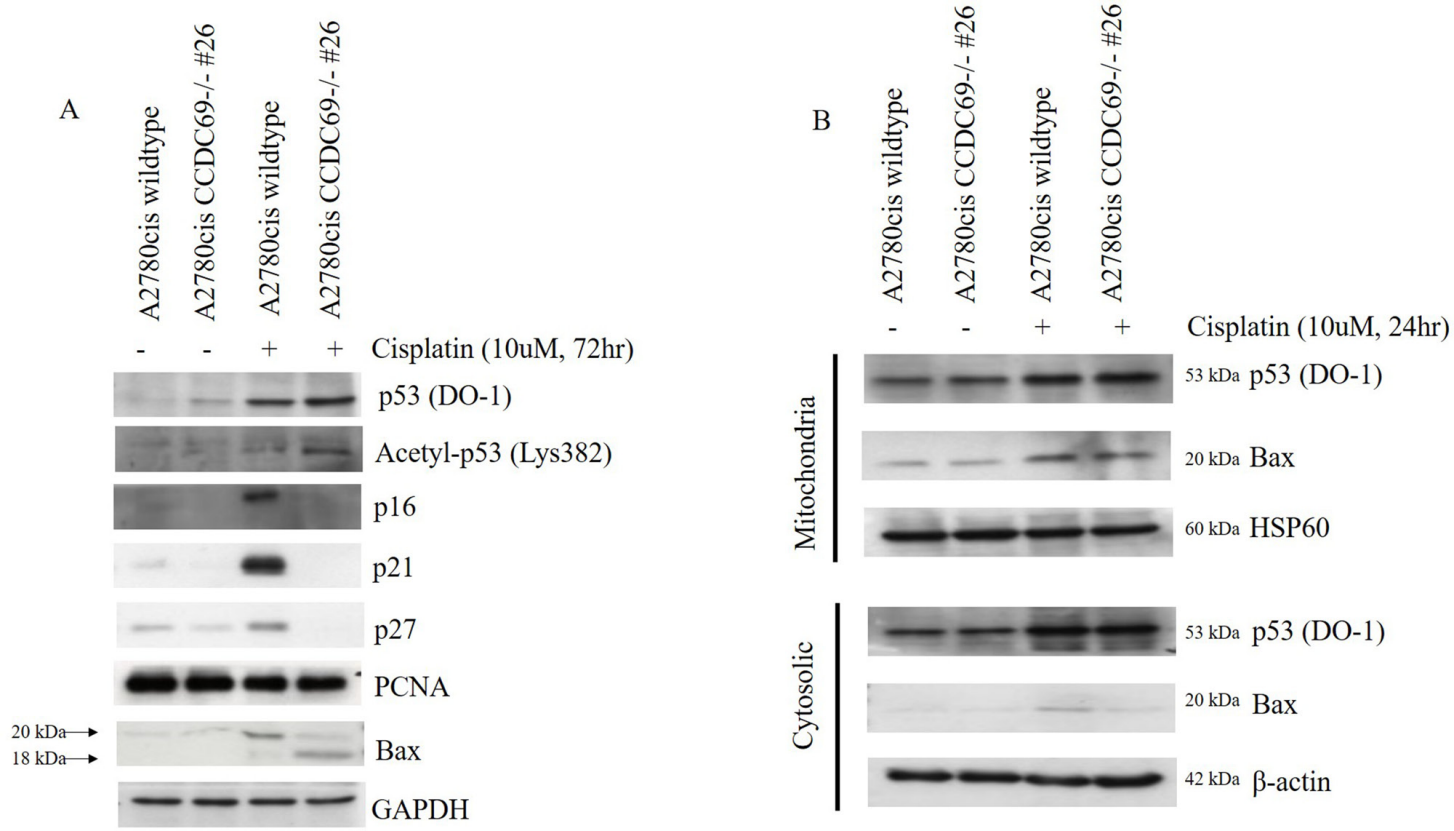
Effect of CCDC69 deletion on expression of p53, acetyl-p53-382 and Bax in response to cisplatin **(A)** The extracted protein samples were analyzed using antibodies against the indicated proteins. Equal protein loading was confirmed by Western blotting for GAPDH. **(B)** After treatment A2780cis wildtype and A2780cis CCDC69-/- #26 cells with 10 μM cisplatin for 24 h, cytosolic and mitochondrial fractions were obtained. The amounts of protein loaded were 20 μg for cytosol and crude mitochondria. HSP60 and β-actin were used as specific marker proteins for mitochondria and cytosol, respectively.

Cyclin-dependent kinase (CDK) inhibitors are negatively responsible for cell cycle progression and cyclin-CDK complexes activity inhibition [30]. To elucidate mechanism behind, we next investigated whether CDK inhibitors, such as p16, p27 and p21, were involved in the cisplatin-induced cell cycle arrest in A2780cis chemoresistant cells by western blotting analysis (Figure 4A). Western blotting results showed that there was a dramatically decrease in expression of p16, p27 and p21 in A2780cis CCDC69-/- #26 cells. These were consistent with abrogation cell cycle arrest in G1 phase in cisplatin resistant A2780cis cells, which could be executed by the CDK inhibitor p21 [31, 32]. On the other hand, the CDK inhibitor p16 is another regulator of cell cycle arrest and senescence [33]. Thus, in the A2780cis CCDC69-/- #26 cells, decreased expression of p16 results in increased CDK4 kinase activity, leading to a complete hyperphosphorylation of the Rb protein. Thus, it is tempting to speculate that reduction of CDK inhibitors after CCDC69 depletion contribute to abrogation of G1 following treatment with cisplatin.

We then asked whether depletion of *CCDC69* altered p53/p21 pathway in A2780cis chemoresistant cells in response to cisplatin. As shown in Figure 4A, although no prominent change was found in the total expression of p53 between both the A2780cis wildtype and A2780cis CCDC69-/- #26 cells following a 72-hour exposure to cisplatin. The upregulation of p21 were more pronounced in A2780cis wildtype cells. Hence, the observed p21 upregulation appeared to be p53-independent.

Recent studies suggested that acetyl-p53-382 is essential for p53 transcription-independent role in Bax activation [19, 34]. To explore combined effects of CCDC69 depletion and cisplatin incubation in cisplatin-induced apoptosis, we evaluated the acetylated p53 (acetyl-p53, Lys 382). As shown in Figure 4A, acetyl-p53-382 levels were marked increased in A2780cis CCDC69-/- #26 cells treated with cisplatin. Our data suggested that CCDC69 depletion and cisplatin incubation act concertedly to increase the acetyl-p53 level compared with A2780cis wildtype cisplatin treated alone. We also performed western blotting assays in A2780cis cells to assess Bax expression in cisplatin-induced apoptosis. Our results showed that, in cells treated with cisplatin, in addition to the full-length Bax, a prominent 18-kDa fragment proapoptotic appeared in A2780cis CCDC69-/- #26 cells (Figure 4A).

Based on the observation that depletion of CCDC69 induced apoptosis, loss of ΔΨm and upregulation of pro-apoptotic Bax. Additionally, previous reports have shown that intrinsic apoptosis signaling activation was associated with Bax mitochondrial translocation in response to apoptotic stimuli [35, 36]. Mitochondria fractionation experiments revealed that Bax was found both in the cytosol and mitochondria in A2780cis wildtype and A2780cis CCDC69-/- #26 cells. By contrast, in A2780cis CCDC69-/- #26 cells, Bax almost completely disappeared from the cytosol (Figure 4B). These results suggest that depletion of *CCDC69* in A2780cis cells induces cisplatin-induced apoptosis via activation of mitochondrial pathway.

### Depletion of CCDC69 enhanced cisplatin-induced apoptosis in ovarian cancer are mediated by c-Myc

**Figure 5:**
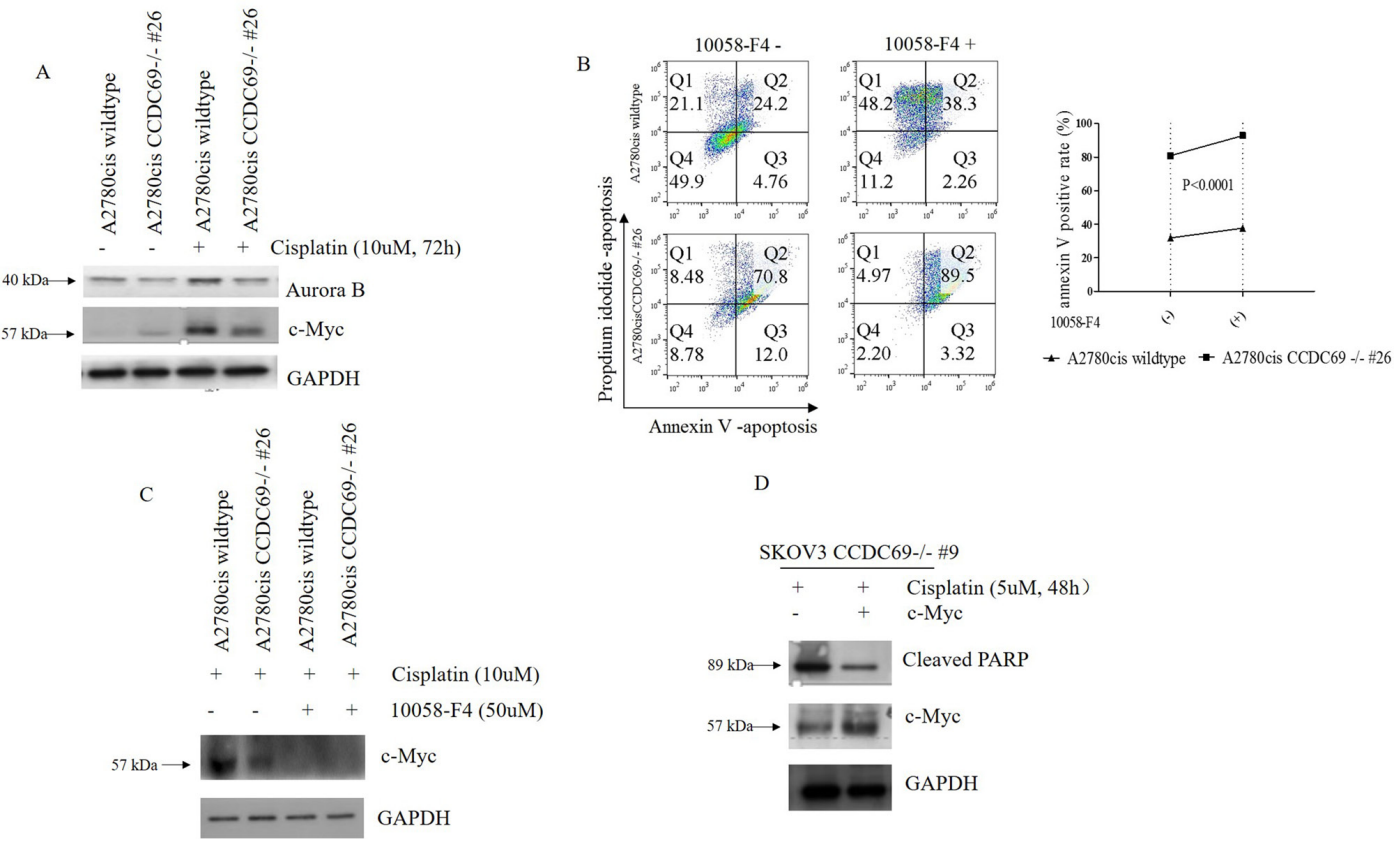
Depletion of *CCDC69* enhanced cisplatin-induced apoptosis in ovarian cancer are regulated by c-Myc **(A)** Western blot showing reduction of Aurora B and c-Myc expression in A2780cis CCDC69-/- #26 cells in response to treatment with cisplatin. **(B)** Flow cytometry analysis of apoptosis in A2780cis wildtype and A2780cis CCDC69-/- #26 cells after after 72 hr cisplatin treatment as well as treatment with c-Myc inhibitor 10058-F4 (50uM). The Annexin v positive rate was analyzed. Data are shown as mean ± s.d. n = 3 independent experiments (^**^: P < 0.01, two-way ANOVA test). **(C)** Western blot showing reduction of c-Myc expression in in A2780cis CCDC69-/- #26 cells in response to treatment with c-Myc inhibitor 10058-F4 (50uM). **(D)** Expression levels of pCMV6-MYC-GFP in SKOV3 CCDC69-/- #9 cells was detected by Western blotting analysis.

It has been shown that Aurora B inhibition in HeLa cells led to the mislocalization of CCDC69 at the central spindle and c-Myc has been shown to mediate Aurora B expression [21, 37]. To identify the involvement of c-Myc and Aurora B after CCDC69 depletion in A2780cis wildtype and A2780cis CCDC69-/- #26 cells, we performed western blotting assays. Consistent with previous studies, cisplatin treatment resulted in upregulation of Aurora B in both cell lines [38, 39]. However, the upregulation of aurora B was more pronounced in A2780cis wildtype cells (Figure 5A). Results also showed c-Myc protein expression was elevated in cisplatin resistant A2780cis wildtype cells compared to the A2780cis CCDC69-/- #26 cells after 72 hr cisplatin treatment (Figure 5A). To know if c-Myc plays a direct role on blocking cisplatin-induced apoptosis, we used the c-Myc inhibitor 10058-F4 to inhibit c-Myc activity by targeting c-Myc-Max interaction. Our data showed that inhibiting c-Myc in A2780cis CCDC69-/- #26 cells had enhanced cisplatin-induced apoptosis in contrast to the A2780cis wildtype cells (P<0.0001, Figure 5B). As shown in Figure 5C, c-Myc expression level was decreased after treatment with 50uM 10058-F4 for 24 hrs (Figure 5C). The c-Myc protein expression level was upregulated in SKOV3 CCDC69-/- #9 cells after transiently transfection with c-Myc plasmid, as supported by western blotting analysis (Figure 5D). We observed that SKOV3 CCDC69-/- #9 cells showed resistant to this cisplatin treatment after c-Myc expression as evident by the induction of apoptosis (Figure 5D). These results suggest that the c-Myc mediated cisplatin induced apoptosis in CCDC69 knockout ovarian cancer cell lines.

### *CCDC69* promoter was heavily methylated in ovarian cancer A2780 and A2780cis cells but not in SKOV3 cells

To know the *CCDC69* expression in human normal ovary tissue and ovarian cancer, we found *CCDC69* expression was significant downregulated in ovarian cancer compared to normal ovary tissue from The Cancer Genome Atlas (TCGA) and Lu et al. database [40] (p<0.001, p<0.05) (Supplementary Figure 4A-4B).

To determine whether downregulation of *CCDC69* expression is associated with DNA methylation, we initially analyzed the statuses of the *CCDC69* methylation in human ovarian cancer in TCGA. The TCGA database showed that there was a significant negatively correlation between *CCDC69* promoter methylation and expression (p=0.0002), as shown in Supplementary Figure 4C. The correlation between copy number alterations and mRNA expression was also examined, but it was not found significantly correlated (p=0.186), as shown in Supplementary Figure 4D.

**Figure 6:**
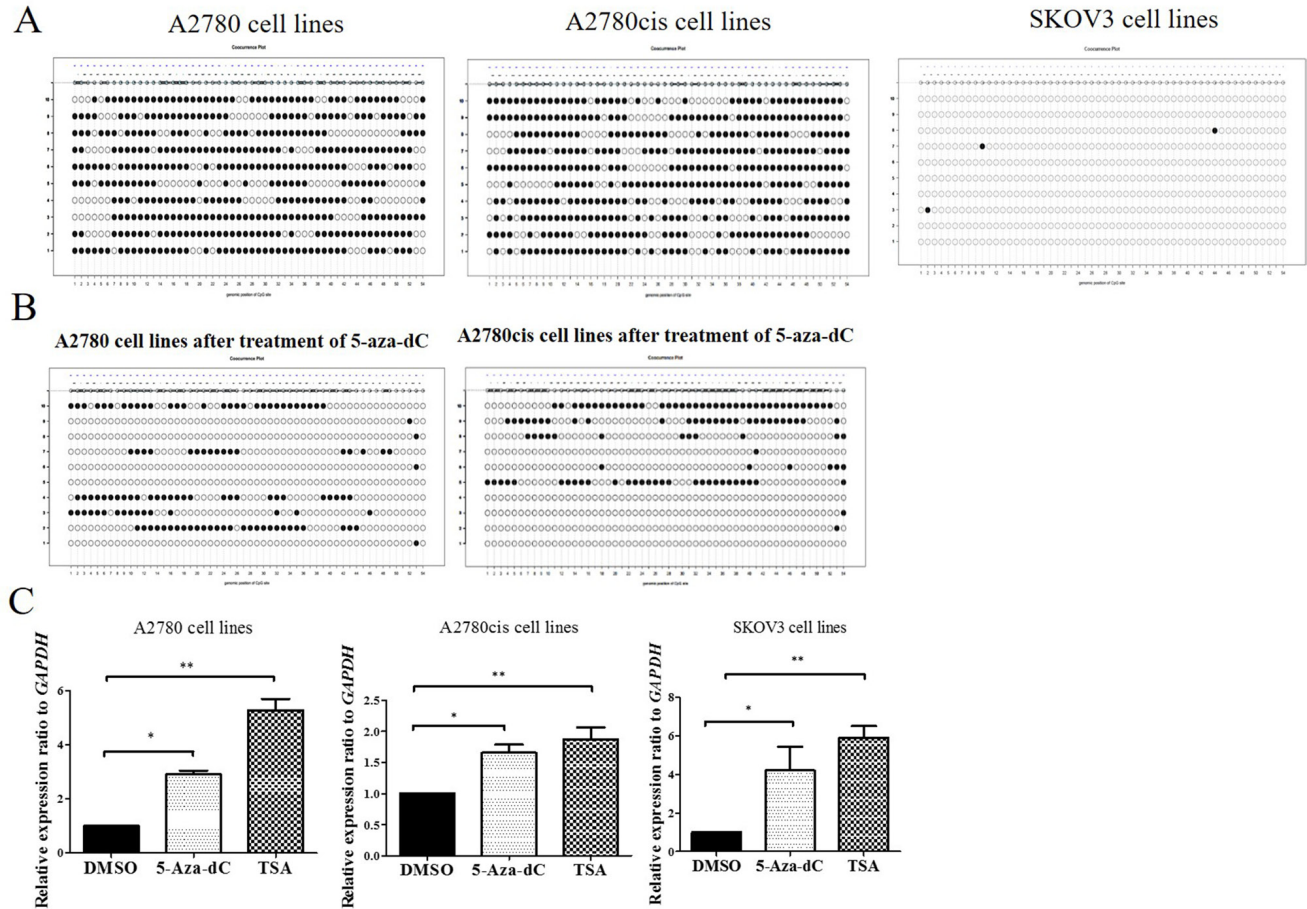
DNA methylation analysis of promoter regions of *CCDC69* in ovarian cancer cell lines **(A)** Representative methylation patterns in promoter regions of *CCDC69* in A2780, A2780cis and SKOV3 ovarian cancer cell lines before treatment with 5-aza-dC. **(B)** Representative methylation patterns in *CCDC69* CpG islands observed in A2780 and A2780cis after treatment with 5-aza-dC. **(C)** Effect of 5-aza-dC and TSA on *CCDC69* expression in A2780, A2780cis and SKOV3 ovarian cancer cell lines. Bisulfite sequencing was performed and the results were visualized by MethVisual [28]. Lollipop displays for each 10 cloning methylation profiles in the genomic context are shown. Filled dots refer to methylated sites, empty dots to non-methylated sites. Each clone of the experiment is displayed separately according to its methylation status. Lengths of the connecting lines correspond to relative genomic distance between CpG sites. Mann–Whitney U-test, (^*^) P < 0.05; (^**^) P < 0.01.

To expand this analysis, we first examined *CCDC69* CpG sites referred to UCSC genome browser, as shown in Supplementary Figure 4E. Then, we found *CCDC69* promoter region encompasses 54 CpG sites by bisulfite sequencing. Heavy CpG methylation (73.1% and 74.3%) was found in A2780 and A2780cis cell lines respectively and rare CpG methylation found in SKOV3 cell lines (Figure 6A).

We also found that demethylation of *CCDC69* CpG sites were significantly decreased treating with 5-Aza-dC in the A2780 and A2780cis cell lines (Figure 6B). This was concomitant with upregulation in the expression of *CCDC69* (p<0.05) (Figure 6C). We also found that *CCDC69* expression was upregulated after treated with TSA in A2780 and A2780cis cells lines. Interestingly, in SKOV3, which showed no methylation, *CCDC69* could be upregulated by 5-Aza-dC and TSA treatment separately. It was found that 5-Aza-dC not only demethylates CpG islands but also alters histone modifications [41]. Taken together, these results imply that repressed expression of *CCDC69* in certain ovarian cancer cell lines may due to both DNA methylation and histone deacetylation.

## DISCUSSION

Several studies have shown that cell cycle modulators have also been identified as mediators of cisplatin resistance [29, 42]. Cells treatment with cisplatin undergoes S and G2 phase arrest in a dose-dependent manner [43]. Actually, a variety of chemotherapy agents including cisplatin that inhibit the G2 phase progression and therefore enhance cisplatin-induced cell death [44, 45]. In our study, our flow cytometry results firstly showed that depletion of CCDC69 abrogated G1 and G2/M phase arrest in cisplatin-resistant A2780cis cells. CCDC69 knockout A2780cis cells exhibited reduction of p21 after exposure to cisplatin. It was reported that p21 not only is involved in maintaining the G1 cell cycle phase arrest in response to DNA damage [46], but also function as an important mediator in G2/M phase through multiple mechanisms in eukaryotic cells [47, 48]. P21 protein inhibits the CDK4/cyclin D1 complexes and thereby maintains the hypophosphorylated repressor state of Rb that blocks transition the cell from G1 to S phase. It was shown that p21 inhibits Cdc2/cyclin B1 complex and G2/M transition even with a weak affinity with the complex [49, 50]. Published work has further shown arrested cells are more resistant to chemotherapy agents than cells lacking of a functional p21 [51]. In our study, CCDC69 worked as a crucial G2/M cell cycle regulator, which upregulated the expression level of Cdc2, together with a prominent increase in cyclin B1 following cisplatin treatment.

In Rb-pathway, p16 not only mediates cell cycle progression through blocking CDK4/ cyclin D1 activity, but also show as a factor maintaining cisplatin resistance in cancer cells [52]. In our study, p16 was marked downregulated after depletion of CCDC69 following treatment with cisplatin. The reduction of p16 can be explained by inactivation of Rb by phosphorylation or the elevated expression of CDK4/cyclin D1 kinase in CCDC69 knockout A2780cis cells. The above results indicates that loss of CCDC69 confers to sensitivity by mitigating G1 and G2/M transition in cisplatin resistant ovarian cancer cells. However, controversial observation regarding the function of p16 on cisplatin sensitivity has been reported [53]. The causal relationship of p16, p21, and CCDC69 expression in chemoresistance remains to be elucidated.

There are two main pathways to apoptotic apoptosis such as extrinsic pathway and intrinsic pathway. In this study, significant activation of both caspase-8 and caspase-3 was found in CCDC69 knockout A2780cis cells following treatment with cisplatin. A significant loss of ΔΨm in CCDC69 knockout A2780cis cells was also identified later. Collectively, these results suggested that both extrinsic and intrinsic pathway was involved in cisplatin induced apoptosis in this study.

It was been well accepted that, Bax protein mainly locates inside the cytosol and translocates to mitochondria membrane during apoptosis in healthy living cells [54]. Induction of apoptosis was occurred while Bax translocated from cytoplasmic to the mitochondria [55, 56]. The present results turned out to have the same theory that translocation of Bax from cytosol to mitochondria was found in CCDC69 depletion cells during cisplatin-induced apoptosis. However, the defined molecular mechanism was still not clear [57]. Bax is transcriptionally activated by p53-dependent apoptosis pathway [58]. However, recent studies have shown that Bax can be activated in a p53 independent manner [59, 60]. Michela Muscolini et al. reported a novel p53 K351N mutation that did not decrease p53 expression, however, the mutation was related to defect in both Bax expression and mitochondrial membrane potential [61]. In our study, we expected the increase in the level of p53 protein of these cells in cisplatin induced DNA-damage. However, we were not able to observe a significant change in p53 protein compared with CCDC69 knockout and wildtype A2780cis cells. Whereas the expression of downstream target genes of p53, p21, p27 and Bax were significantly increased, indicating that p53 was activated in A2780cis cells. Our data showed that the increased acetylation of p53 at K382 sites in CCDC69 depleted A2780cis cells. Previous study has demonstrated that p53 acetylation is responsible for disruption of the Ku70-BAX complex, leading to Bax conformational alteration and translocation [19]. It implies that p53 acetylation at K382 may contribute to enhance cisplatin-induced apoptosis after depletion of CCDC69. However, further mechanism studies should be put forward in the future.

Studies have demonstrated that Aurora B inhibitors sensitized cisplatin-resistant ovarian cancer cells but its underlying mechanism are still not fully understood [38, 62, 63]. It was showed that combination treatment efficacy of Aurora B inhibitor and cisplatin is related to c-Myc expression [38]. Here we found that inhibition of c-Myc enhanced sensitivity of cisplatin-induced apoptosis in CCDC69-depleted A2780cis cells, overexpression of c-Myc reduce apoptosis in CCDC69 knockout SKOV3 cells. In consistent with previous report [21], we found marked decreased expression of Aurora B after depletion of CCDC69 in A2780cis cells following treatment with cisplatin. Gully et al. showed that Aurora B phosphorylates p53 to promote rapid degradation of p53 by ubiquitination mechanisms [39]. However, we observed a slightly higher expression of p53 instead of decreased expression. It is implying that other molecular pathway may be activated via loss of CCDC69 to offset the degradation of p53.

In addition to finding evidence of CCDC69 silencing led to cisplatin sensitivity in ovarian cancer cells, the bisulfite sequencing data showed that CCDC69 promotor region was heavily methylated in A2780 and A2780cis ovarian cancer cells. These results were firmly confirmed by restoration experiments. Histone deacetylase (HDAC) inhibitor, TSA, not only restored CCDC69 gene expression in hypermethylated A2780 and A2780cis cells, but also increased its expression in hypomethylated SKOV3 cells. As it was demonstrated that, both DNA methylation and histone deacetylation may be responsible for mediating CCDC69 gene expressions in ovarian cancer cells. However, the amount of examined cell lines were too small to give rise to confirmative conclusion on the effect of 5-aza-dC and TSA. In addition, CCDC69 mRNA expression level was found to be downregulated in cancers of the ovary, breast, esophagus and small intestine compared with corresponding normal tissues as shown in public database (Supplementary Figure 5). These results suggest that CCDC69 may be related to a variety of cancer types including ovarian cancer.

Although epigenetic therapeutic agents has been applied to treat ovarian cancer and other cancers [64–66], we did not observe significant difference in methylation status between cisplatin-sensitive A2780 and cisplatin-resistant A2780cis ovarian cancer cells. The results indicate that the methylation status of the CCDC69 promoter may not be related to responsiveness to cisplatin in these cell lines. Considering that the histone deacetylase (HDAC) inhibitor, TSA, enhanced higher expression of CCDC69 in both hypermethylated A2780 and A2780cis cells, it indicates that transcriptional silencing might be predominantly mediated by histone deacetylation. Previous studies have demonstrated that treatment with HDAC inhibitors may increase the accessibility of DNA to chemotherapeutic agents and induce apoptosis in various cancer cell lines [67, 68]. We postulated that the cancer cells with low expression of CCDC69 under combined treatment with cisplatin and HDAC inhibitor would further increase the efficacy of cisplatin treatment.

In the present study, CRISPR/Cas9 technology was applied to knock out *CCDC69* in ovarian cancer A2780cis and SKOV3 cells. In eukaryotic cells, two repair pathways are active. One is non-homologous end joining, abbreviated NHEJ. The NHEJ can repair double-stranded break (DSB) at any time during cell cycle progression. The other is homology-directed repair (HDR) pathway [27]. However, the HDR shows a relatively low efficiency compared with the NHEJ. Notably, in our study, one double-strand break (DSB) caused by one gRNA in A2780cis CCDC69-/- #26 cells using the CRISPR/Cas9 system led to the same length deletion in both alleles and repaired by HDR. The other SKOV3 CCDC69-/- #9 cells caused by one gRNA and repaired by NHEJ, which results in indels. Therefore, we speculate that CRISPR/Cas9-mediated DSBs at the same sites in both alleles may be repaired by NHEJ pathway due to HDR low efficiency. However, there are limitations in assessing the chemotherapy response using the established ovarian cancer cell lines, which cannot truly reflect the complexity and heterogeneity of tumors in patients. Additional studies of *in vivo* mouse xenograft or patient-derived xenograft (PDX) models derived from ovarian cancer are necessary to evaluate the role of CCDC69 in ovarian cancer, thus allowing better prediction of a patient's chemotherapic outcome [69].

In conclusion, we provide the first evidence that the depletion of CCDC69 reversed platinum resistance in ovarian cancer cells. CCDC69 expression levels may also serve as a predictive marker of responsiveness to chemotherapy. *In vivo* model of targeting CCDC69 and clinical setting of measuring CCDC69 expression in circulating tumor cells are needed for additional in-depth studies.

## MATERIALS AND METHODS

### Reagents

Cisplatin, carboplatin, and c-Myc inhibitor 10058-F4 were purchased from Sigma-Aldrich Co. (St. Louis, MO, USA). Cisplatin and carboplatin were dissolved in 0.9% sodium chloride and dimethyl sulfoxide (DMSO), respectively. 5-aza-2’-deoxycytidine (5-aza-dC; Sigma-Aldrich) and Trichostatin A (TSA, Sigma-Aldrich) were solubilized in DMSO.

### Cell lines

The human ovarian cancer cell line A2780 and its cisplatin-resistant variant A2780cis were obtained from Sigma-Aldrich. To maintain the cisplatin-resistance of A2780cis cells, 1μM cisplatin was added to the culture medium every 2-3 passages. Human ovarian cancer SKOV3 cell lines were provided by Dr. Joseph Kwong. Cells were cultured in high-glucose DMEM (Invitrogen) containing 10% fetal bovine serum (FBS) (Invitrogen) and 100U/ml of penicillin G/streptomycin. These cells were cultured in an incubator at 37 °C in 5% CO2 with a humidified atmosphere.

### Establishment and validation of A2780cis CCDC69-/- #26 and SKOV3 CCDC69-/- #9 cells

To create homologous recombination (HR) assays, gRNAs were designed using the CRISPR design tool (crispr.mit.edu) [27] [26] [26]. The targeted gRNA expression oligos and homology donor template DNA were synthesized by Blue Heron Biotechnology, Origene (Bothell, WA, USA). The sequences of these oligos are shown in Figure 1. Approximately 24 hours before transfection, plate 1×10^5^ A2780cis cells in 2 ml culture medium into each well of a 6-well plate. A mixture of 1 μg of pCas-Guide vector containing each target gRNA and 1 μg of donor DNA was transfected into adherent A2780cis cells. 48 hrs post transfection, transfected cells were cultured in medium containing 2.0 μg/ml puromycin for 3 days for selection. Surviving cells were trypsinized and diluted in medium for colony formation. Single colonies were selected, and each colony was passaged and genotyped. DNA was isolated using a DNeasy Blood & Tissue Kit (Qiagen). The genomic region surrounding the CRISPR/Cas9 target site was PCR amplified, and the amplicons were cloned into the pCR^®^ 4 TOPO^®^ Vector (Invitrogen). Each colony was selected, and the amplicon sequences were analyzed using a 3100 Genetic Analyzer (ABI). Supplementary Table 1 shows the primer sequences.

### Plasmid and transfection

The eukaryotic expression plasmid pCMV6-CCDC69-GFP and pCMV6-MYC-GFP containing the wild-type CCDC69 (NM_015621, cat# RG217758) and MYC (NM_002467, cat# RG201611), respectively. All these vectors including the empty plasmid pCMV6-AC-GFP (cat# PS100010) were from Origene (Bothell, WA, USA). The day before transfection, cells were seeded in a 6-well plate at a concentration of 1×10^5^ cells per well. Transfection was performed using Lipofectamine^TM^ 2000 (Invitrogen) according to the manufacturer's instructions. The empty vector was used as negative control.

### Cell viability assay

Cells were seeded in 96-well plate at a density of 5×10^3^ cells/well. After an overnight growth, cells were treated with cisplatin. After treatment for indicated time courses, cell viability was measured by MTT assay using the Cell Counting Kit-8 (CCK-8, Sigma-Aldrich) following the manufacturer's instructions. The absorbance at 450 nm was measured in the microplate reader. The percentage of cell survival at each dose of cisplatin = Mean of A450 (drug-treated cells)/Mean of A450 (untreated cells). IC50 values were calculated using GraphPad Prism Software Version 5 (GraphPad Software Inc., CA, USA) and plotted in dose response curves.

### Flow cytometry analysis of cell apoptosis using Annexin V-FITC/PI staining

Cells were either kept untreated or exposed to cisplatin for indicated time before analysis by flow cytometry. The detection was performed according to the manual of Alexa Fluor^®^ 488 Annexin V/Dead Cell Apoptosis Kit (Invitrogen). About 1×10^6^ cells were collected, washed with ice-cold PBS, and resuspended in binding buffer containing suitable amount of Annexin V-FITC. After 15 min of incubation in the dark at room temperature, the buffer was removed by centrifugation. The cells were then resuspended in reaction buffer containing propidium iodide (PI). Flow cytometry analysis was performed immediately to detect apoptosis.

### Mitochondrial membrane potential assay

The mitochondrial membrane potential (ΔΨm) was detected according to the manual of The MitoProbe™ JC-1 Assay Kit (Invitrogen). Briefly, cells suspended in 1 ml PBS at approximately 1×10^6^ cells/ml, were incubated with 2 μM of JC-1 for 15 minutes at 37°C. The cells were washed and resuspended in 500 μl PBS and then analyzed on a flow cytometer with 488 nm excitation and emission at 590 nm (red) and 540 nm (green). A decrease in the red/green fluorescence intensity ratio was interpreted as a loss of ΔΨm, whereas an increase in the ratio was interpreted as a gain in ΔΨm.

### Caspase activity assay

Following cisplatin treatments, cells were subjected to Caspase 3/7 activities measurement with Caspase-Glo assay kit from Promega (Madison, WI, USA). Briefly, the plates containing cells were removed from the incubator and allowed to equilibrate to room temperature for 30 minutes. 100 μl of Caspase-Glo reagent was added to each well, the content of well was gently mixed with a plate shaker at 300–500 rpm for 30 seconds. The plate was then incubated at room temperature for 2 hours. The luminescence of each sample was measured in a plate-reading luminometer (PerkinElmer). The experiments were performed in triplicate and repeated on two separately-initiated cultures. Cleaved caspase 8 was assessed by flow cytometry. Briefly, cells were fixed with 70% ethanol for 10 minutes, permeabilized with 0.25% Triton™ X-100 for 20 minutes, and blocked with 5% BSA for 30 minutes at room temperature. The cells were then incubated with cleaved caspase 8 (Asp391) at 1/100. The secondary antibody used was goat anti-rabbit IgG (488 FITC) at 1/200 (2.5ul/500ul) dilution. The representative 10,000 cells were acquired using a laser (488 nm) and 530/30 bandpass filter and analyzed for each sample using Cytomics FC 500 (Beckman Coulter) flow cytometer.

### Colony formation assay

Cells were plated in 6-well plates with 1 × 10^3^ cells per well in duplicate and pretreated with 10 μM Cisplatin for 48 hrs. Then, the cells were washed out and incubated at 37 °C in growth medium for 14 days. These colonies were fixed with 4% paraformaldehyde for 15 minutes and stained with crystal violet for five minutes. The colonies were counted from three independent experiments using Image J software and plotted as mean ± SE.

### Cell cycle assay

After the indicated treatments, cells were washed with cold PBS and harvested by centrifugation. Then, cells were re-suspended in 70% (v/v) cold ethanol and stored at −20 °C overnight. After 30-minute incubation with propidium iodide (PI) solution in the dark, cell cycle distribution was analyzed by Cytomics FC 500 (Beckman Coulter) flow cytometer. Results were calculated and visualized by Flow Jo software (version 10.0).

### Preparation of subcellular fractions

Cells were pretreated with cisplatin and then incubated for different durations. Untreated cells were used as control. Subcellular fractions were separated according to the manual of Mitochondria/Cytosol Fractionation Kit (Abcam). Cell lysates were homogenised using a dounce homogeniser (50 strokes/sample). After an incubation on ice for 30 min, the unbroken cells and nuclei were pelleted by centrifugation at 2500 g for 10 min. The supernatant was collected and further centrifuged at 13,000 g for 15 min. The pellet containing the mitochondrial fraction was then resuspended in supplied buffer. P53 and Bax protein levels in the mitochondrial fraction were then determined. The remaining supernatant was further centrifuged at 100,000 g for 1 h. The final supernatant was designated as the cytosolic fraction, which was used for detection of Bax.

### Protein extraction and western blotting

Total protein was extracted with RIPA lysis buffer containing 1% protease inhibitor cocktail (Abcam). Equal amounts of total protein were subjected to sodium dodecyl sulfate-polyacrylamide gel electrophoresis (SDS-PAGE) and were transferred onto a polyvinylidene fluoride (PVDF) membrane. Blocking for one hour was at room temperature with 5% non-fat milk. The membrane was incubated overnight with the primary antibodies (dilution ration 1:1000) at 4 °C with at room temperature, including CDK4, Cyclin D1, Phospho-Rb (Ser780), p27, p21, c-Myc, p16, p-Cdc2 (Try15), Cdc2, Cyclin B1, Acetyl-p53 (Lys382), Bax and Hsp60, and the incubation for the secondary antibodies was one hour at room temperature. Bands visualization was conducted by an ECL Advance Western Blotting Detection Kit (Amersham, UK). Antibodies against CCDC69, c-Myc and p16 were from Abcam (ab175301, ab32072 and ab108349). p53 Antibody (DO-1) antibody was purchased from Santa Cruz Biotechnology (Santa Cruz, CA, USA). All other antibodies were purchased from Cell Signaling Technology (Danvers, MA, USA). An HRP-conjugated anti-rabbit IgG antibody was used as secondary antibody (Abcam, ab191866). β-actin or GAPDH was used as an internal control for normalization of protein quantity.

### Sodium bisulfite modification and bisulfite sequencing

Each genomic DNA (1 μg) was modified by sodium bisulfite using the EZ DNA Methylation Kit (Zymo Research, Orange, CA, USA) according to the manufacturer's instructions. To perform bisulfite sequencing analysis on the 5’-upstream region of CCDC69 (CpG sites from −272 to +368), 25 ng bisulfite-modified DNA was amplified in a 20 μl reaction with forward primer 5’- AGAAGGATTTTGGTTATTTGT -3’ and reverse primer 5’- TCACTTATCCATAACCACACA -3’ (designed using MethPrimer, http://www.urogene.org/methprimer/index.html) to yield a 640 bp product. PCR products were cloned into TOPO® TA cloning Vector [Invitrogen]), and 10 white colonies were randomly chosen for sequencing. DNA methylation data were visualized by R package ‘methVisual’ [28].

### Restoration of CCDC69 expression by treatment with 5-aza-2’-deoxycytidine

Ovarian cancer cell lines (A2780, A2780cis and SKOV3) were seeded in 35 mm dishes at a density of 1×10^5^ cells/dish 1 day before drug treatment. Cells were treated with 10 μM 5-aza-dC every 24 h for 3 days and then harvested. Another culture of cells was treated with 0.5 μM TSA for 1 day. DNA was prepared and tested for reversion of CCDC69 methylation by bisulfite sequencing. Total RNA was prepared and tested for restoration of CCDC69 expression by real-time PCR.

## SUPPLEMENTARY MATERIALS FIGURES AND TABLE


