# Supplementary material for: Inhibition of coiled coil domain containing protein 69 enhances platinum-induced apoptosis in ovarian cancer cells
Source: Oncotarget. 2017 Sep 28;8(60):101634–48. doi: 10.18632/oncotarget.21356 (PMC5731902; doi:10.18632/oncotarget.21356)
Supplement: Supplementary file 1 [file oncotarget-08-101634-s001.pdf]

Inhibition of coiled coil domain containing protein 69 enhances platinum-induced apoptosis in ovarian cancer cells

SUPPLEMENTARY MATERIALS

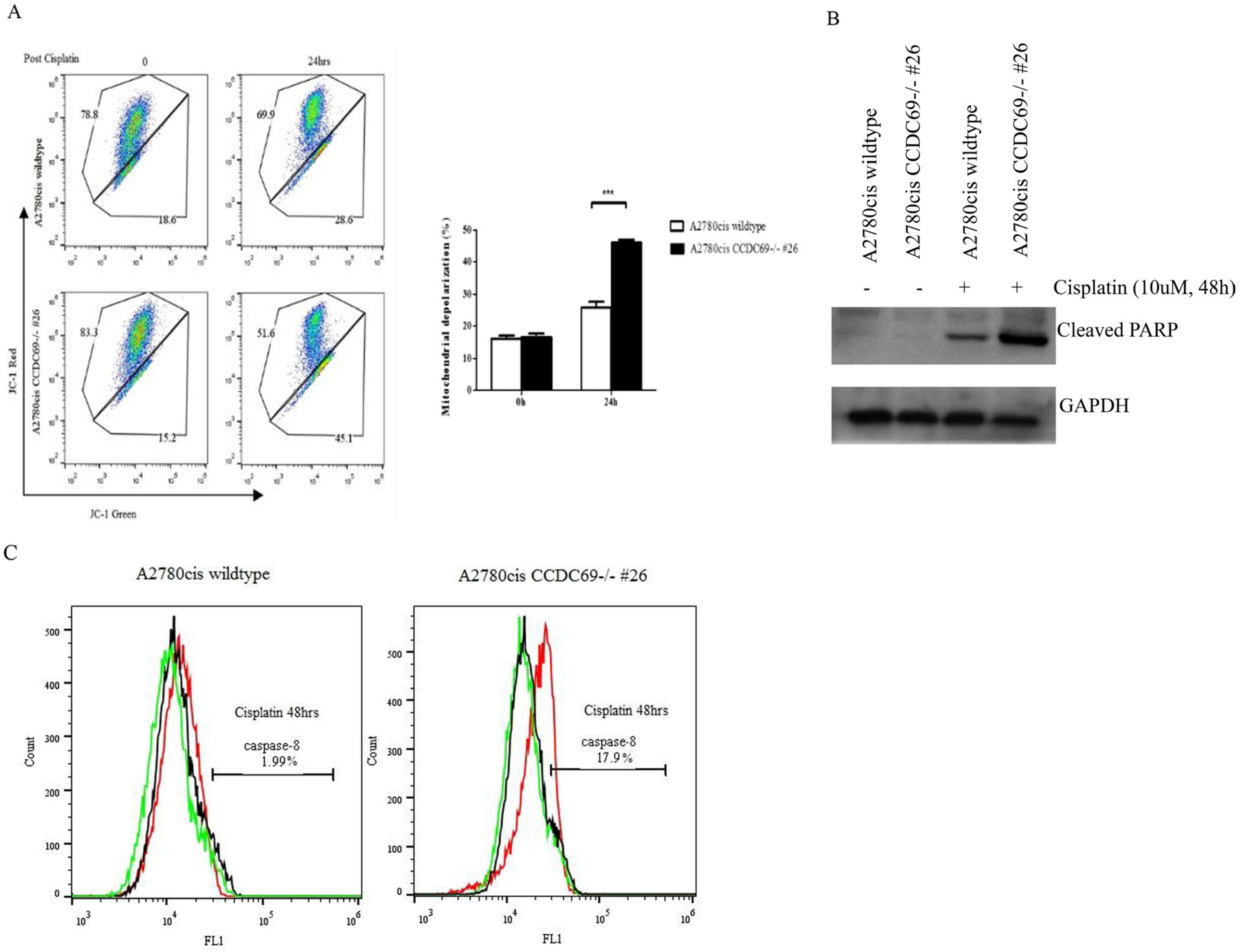

Supplementary Figure 1: Depletion of *CCDC69* in ovarian cancer cells enhanced cisplatin induced-apoptosis. (A) Scatter plots of JC-1 staining for mitochondria membrane potential as measured through JC-1 staining and quantification of cells with depolarized mitochondria, which is indicative of mitochondria membrane depolarization. **(B)** Increase cleavage PARP activity after treatment with cisplatin. Immunoblot analysis of cleavage PARP in A2780cis wildtype and A2780cis CCDC69-/- #26 cells after exposure to cisplatin. **(C)** Increase cleavage caspase 8 activity after treatment with cisplatin. Flow cytometric analysis for the expression rate of caspase-8 after cisplatin (10 µM) stimulation for 48 hrs. Unlabelled control (black line) was cells without incubation with primary antibody and secondary antibody. Untreated samples (green line) and cisplatin treated samples (red line) were cells incubated with cleaved caspase 8 (Asp391) and goat anti-rabbit IgG (488 FITC). Bars for all graphs represent the mean of at least three independent experiments. (*) $P < 0.05$; (**) $P < 0.01$; (***) $P < 0.001$.

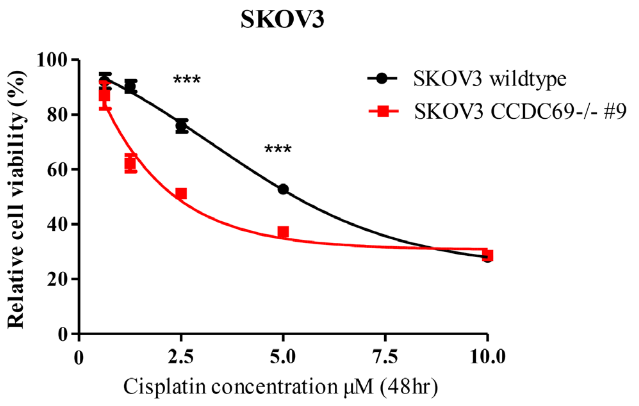

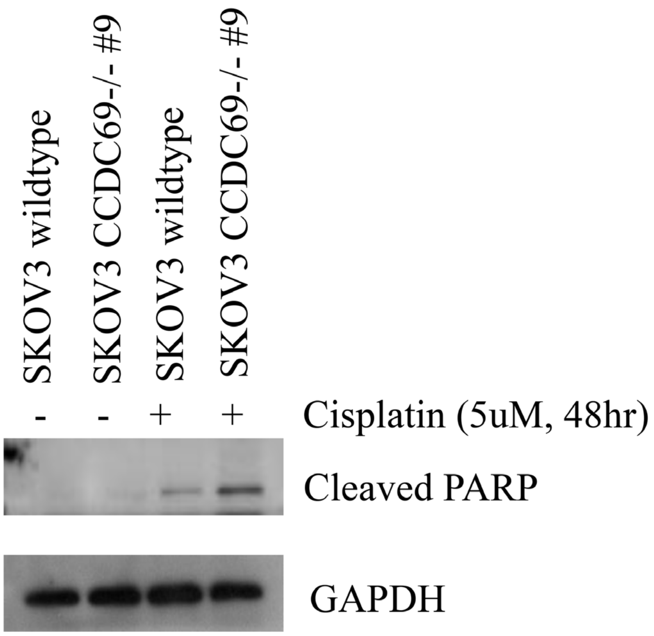

Supplementary Figure 2: Depletion of *CCDC69* in SKOV3 ovarian cancer cells enhanced induced cisplatin induced apoptosis. Effect of cisplatin on cell viability. SKOV3 cells were treated with different concentrations of cisplatin for 48 h. Cell viabilities were assessed by CCK8 assay. Apoptosis of *CCDC69* knockout SKOV3 cells were detected by cleavage of PARP. The percent cell viabilities were calculated by normalizing with a control panel. Data are representative of six independent trials and are expressed as the mean±SD. Columns, means of data (n=6); bars, SD; **P<0.01, ***P<0.001 relative to the control.

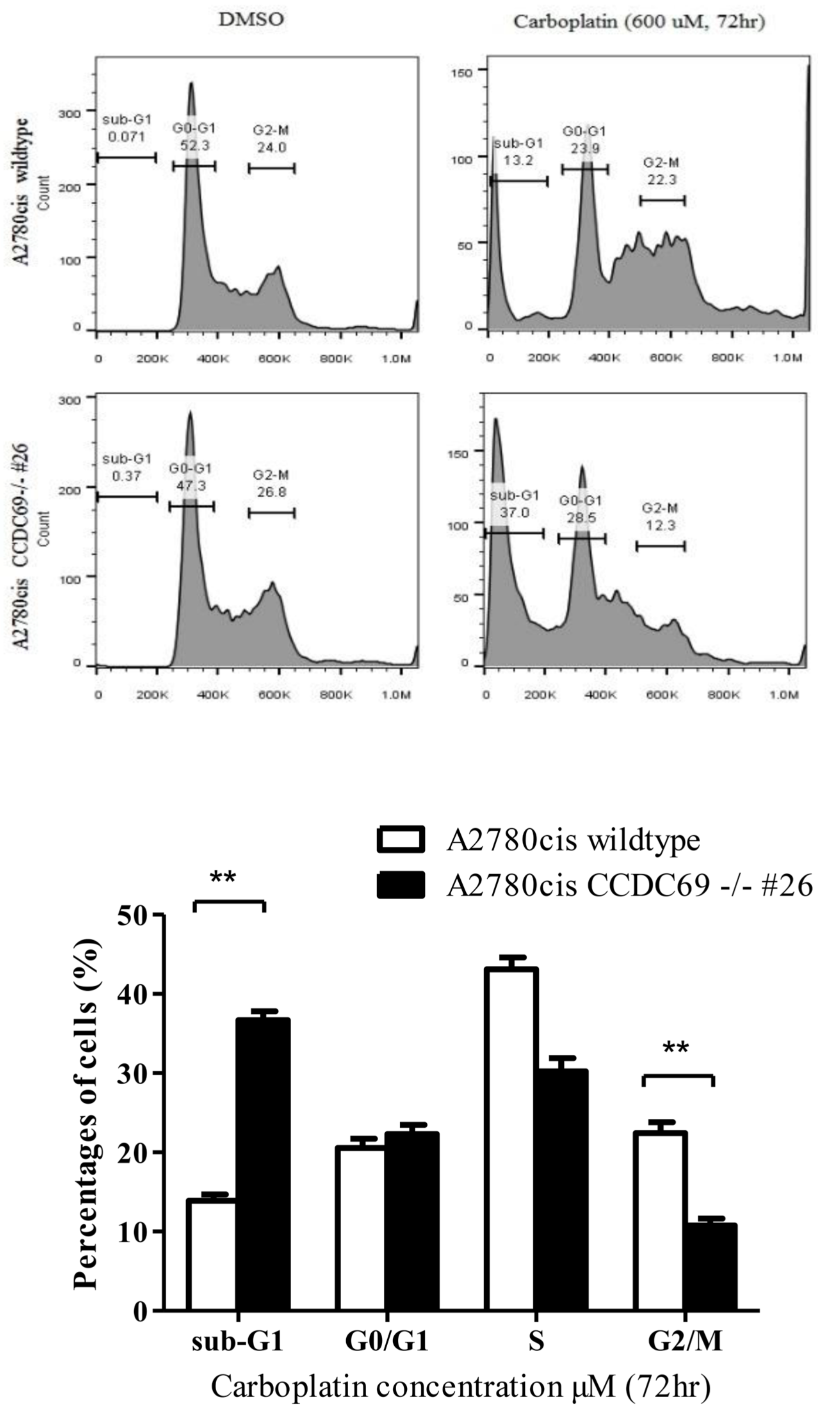

Supplementary Figure 3: *CCDC69* depletion in A2780cis cells abrogated G2/M arrest following carboplatin treatment. Percentages of carboplatin-treated A2780cis CCDC69 -/- #26 cells in G2/M phase were decreased compared to A2780cis wildtype cells, while percentage of treated A2780cis CCDC69 -/- #26 cells in sub-G1 phase was increased. Data represent the mean and the standard deviation from three independent experiments. $^{*}p < 0.05$ and $^{**}p < 0.01$ versus carboplatin-treated A2780cis wildtype cells (Student's t-test).

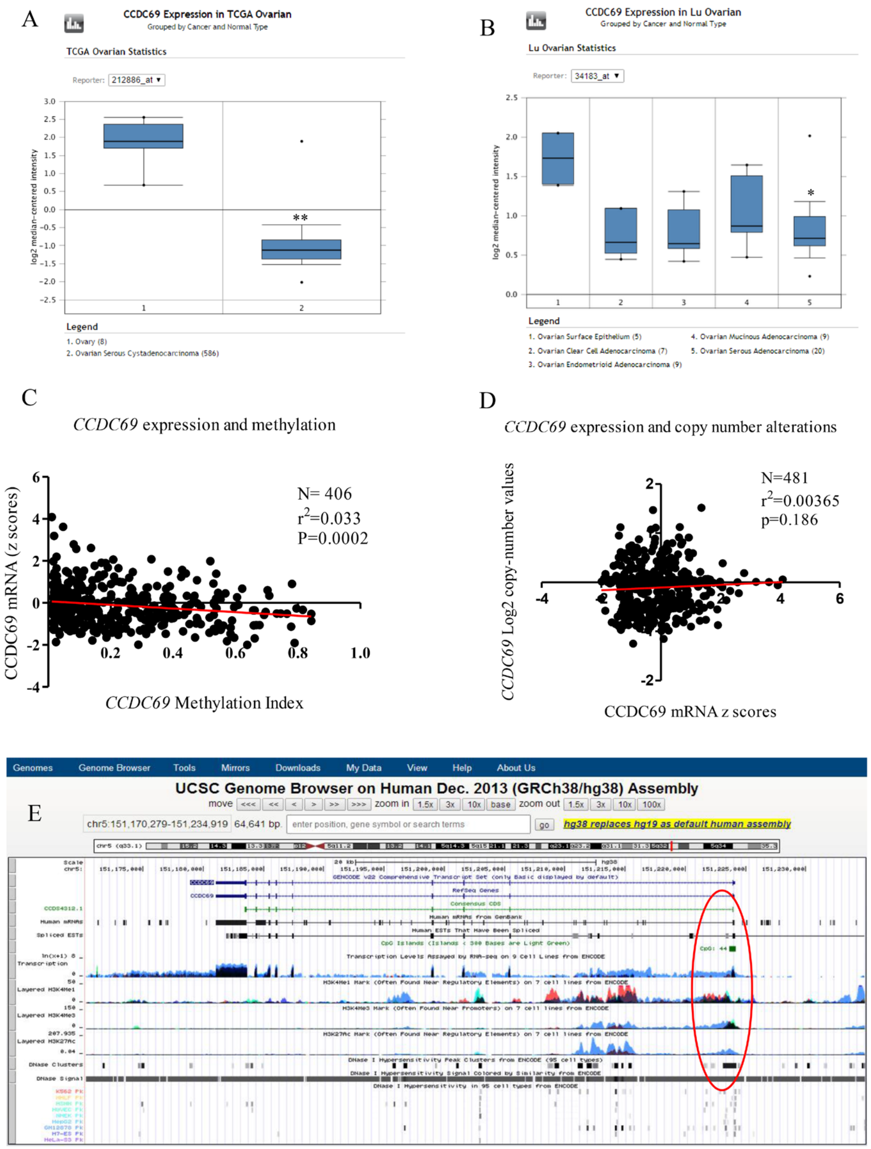

Supplementary Figure 4: *CCDC69* expression and methylation statuses in human ovarian cancer. **(A-B)** *Oncomine* analysis of *CCDC69* expression in human normal ovary tissue and ovarian cancer in TCGA (**p-value < 0.001) and Lu et al. (*p-value < 0.05). Data are presented as a box and whiskers plot, with the box indicating the 25th to 75th percentiles, whiskers indicating the 10th and 90th percentiles, top and bottom points indicating the range, center line indicating the median, and the title shows the referenced studies [40] (http://cancergenome.nih.gov/). **(C)** Correlation analysis of *CCDC69* methylation (x-axis) and expression (y-axis) in the Ovarian Serous Cystadenocarcinoma TCGA, Provisional dataset (n=406) (p=0.0002). **(D)** Analysis of *CCDC69* copy number alterations (CNA) against *CCDC69* expression (z-score) were plotted in the Ovarian Serous Cystadenocarcinoma TCGA, Provisional dataset (n=481) (p=0.186). Copy number was determined by cBioPortal using the GISTIC algorithm. **(E)** Regional DNA methylation in *CCDC69* promoter exon1, intron1, CpG Island. Screenshot of *CCDC69* was taken from the UCSC genome browser (http://genome.ucsc.edu) and it showed the positions of the coding regions and a CpG island containing 54 CpG sites. Red circle indicates the methylated CpG sites.

Supplementary Table 1: Primers sequences flanking CRISPR/Cas9 targeted CCDC69 genomic regions

	Sequence (5’->3’)	Template strand	Length	Start	Stop	Tm	GC%	Self 5’ complementarity	Self 3’ complementarity
69F	TTTTTCCCCCGGCCCTTTC	Plus	19	258	276	60.53	57.89	4.00	0.00
69R	GAGCTGGCCTCTCCAGGTTC	Minus	20	792	773	62.25	65.00	4.00	1.00
Product length				535					

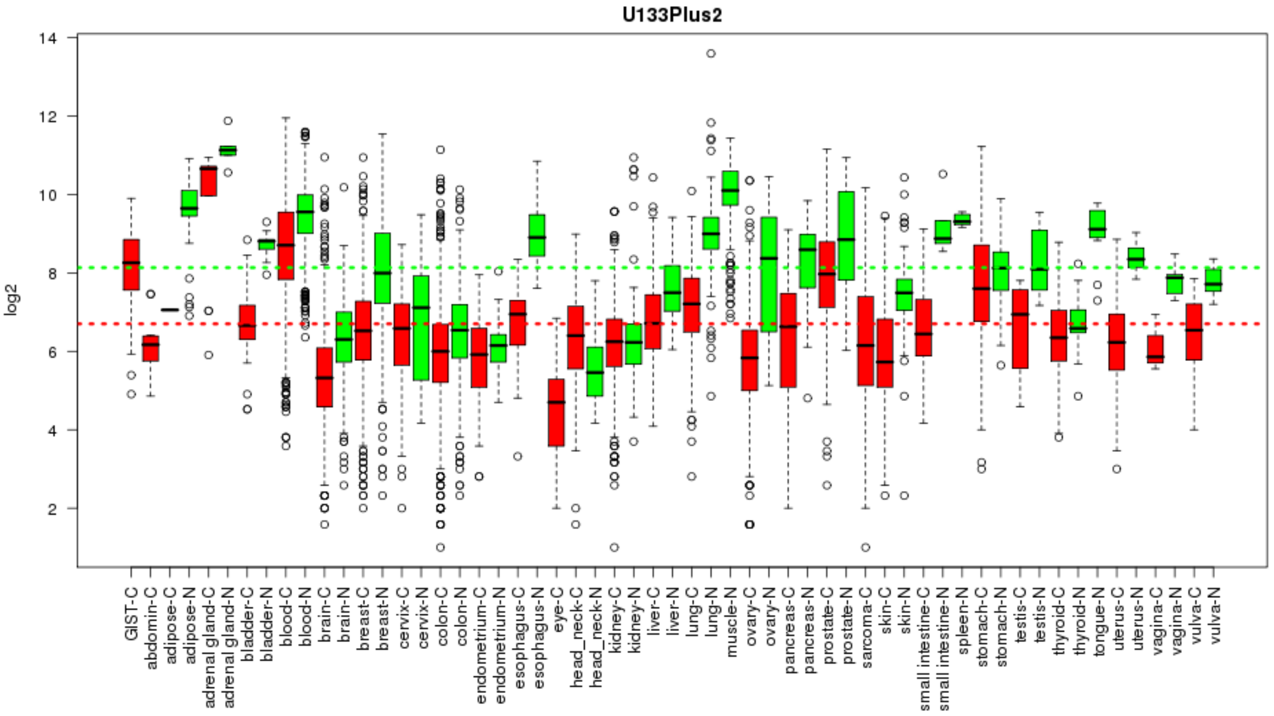

Supplementary Figure 5: Expression pattern of *CCDC69* in normal and tumor tissues. *CCDC69* expression in various types of cancer was searched in the GENT database (available at http://medical-genomics.kribb.re.kr/GENT/). Boxes represent the median and the 25th and 75th percentiles; dots represent outliers. Red boxes represent tumor tissues; green boxes represent normal tissues. Red and green dashed lines represent the average value of all tumor and normal tissues, respectively. The asterisk indicates the significant decrease of *CCDC69* expression in ovarian tumors compared with normal tissues.
